# Supplementary material for: Correct setup of the substantia nigra requires Reelin-mediated fast, laterally-directed migration of dopaminergic neurons
Source: eLife. 2019 Jan 28;8:e41623. doi: 10.7554/eLife.41623 (PMC6349407; doi:10.7554/eLife.41623)
Supplement: Transparent reporting form [file elife-41623-transrepform.docx]

***eLife’s* transparent reporting form**

We encourage authors to provide detailed information *within their submission* to facilitate the interpretation and replication of experiments. Authors can upload supporting documentation to indicate the use of appropriate reporting guidelines for health-related research (see [EQUATOR Network](http://www.equator-network.org/%20)), life science research (see the [BioSharing Information Resource](https://biosharing.org/)), or the [ARRIVE guidelines](http://www.plosbiology.org/article/info:doi/10.1371/journal.pbio.1000412) for reporting work involving animal research. Where applicable, authors should refer to any relevant reporting standards documents in this form.

If you have any questions, please consult our Journal Policies and/or contact us: [editorial@elifesciences.org](mailto:editorial@elifesciences.org).

**Sample-size estimation**

- You should state whether an appropriate sample size was computed when the study was being designed
- You should state the statistical method of sample size computation and any required assumptions
- If no explicit power analysis was used, you should describe how you decided what sample (replicate) size (number) to use

Please outline where this information can be found within the submission (e.g., sections or figure legends), or explain why this information doesn’t apply to your submission:

We did not use power analysis, but based on the robustness of the phenotype in *Dab1* CKO and *Dab1^-/-^* animals, we estimated that n (biological replicates) >=3 would suffice for our analysis.

**Figure 1:** Direct role of Reelin signaling: we used n>= 4 animals for each genotype as the phenotype was very robust and discernible. Number of mice per genotype is stated in the **Figure legend.**

**Figures 2-9:** Rationale for tracking a large population of neurons along with a full description of how the population of cells were divided into subgroups is also provided in **Materials and methods: Speed and trajectories of migrating mDA neurons** (pg. 24; lines 674 – 692).

**Figure 10:** Immuno blot sample size of n =3 or n = 5 animals/genotype was used based on previous studies that reported significant differences with similar sample sizes with the same proteins of interest (Jossin and Goffinet, 2007). Sample sizes are mentioned in figure legends of **Figure 10 and Figure 10 – Figure supplement 1.**

**Replicates**

- You should report how often each experiment was performed
- You should include a definition of biological versus technical replication
- The data obtained should be provided and sufficient information should be provided to indicate the number of independent biological and/or technical replicates
- If you encountered any outliers, you should describe how these were handled
- Criteria for exclusion/inclusion of data should be clearly stated
- High-throughput sequence data should be uploaded before submission, with a private link for reviewers provided (these are available from both GEO and ArrayExpress)

Please outline where this information can be found within the submission (e.g., sections or figure legends), or explain why this information doesn’t apply to your submission:

Slices/sections/protein samples analyzed are biological replicates. We provide information on sample size in each figure legend (where applicable) and in the Materials and Methods.

**Figure 1:** Details of sample sizes for each embryonic/adult time point are provided in figure legends for **Figure 1; Figure 1- Figure supplement 3** and in **Materials and methods: Statistical analysis** (pg. 25; lines 724 – 733).

**Figures 2-7:** We tracked a large number of neurons (806 control; 844 *Dab1^-/-^*) across 3 slices/genotype. Slices were obtained from 4 separate litters. Only slices with well-defined fluorescent cells and no drift were included into the analysis (described pg. 23; line 623 - 624). Sample sizes of subpopulations of fast, moderate and slow neurons are provided in the figure legends of all main and supplementary figures. A full description of how the population of cells were divided into subgroups is also provided in **Materials and methods: Speed and trajectories of migrating mDA neurons** (pg. 24; lines 674 – 692).

**Figure 8 -9:** Morphological analysis was restricted to a subset of tracked neurons (n = 150 control; n = 129 *Dab1^-/-^* neurons) as the analysis was carried out manually and only on neurons with clear distinguishable processes. Sample sizes are mentioned in all applicable figure legends of main and supplementary figures. A full description of sample sizes in these experiments is provided in **Materials and methods: Morphology analysis of migrating mDA neurons** (pg. 24; lines 705 – 722).

**Figure 10:** Immuno blot sample size of n =3 or n = 5 animals/genotype are mentioned in figure legends of **Figure 10; Figure 10 – Figure supplement 1.**

**Statistical reporting**

- Statistical analysis methods should be described and justified
- Raw data should be presented in figures whenever informative to do so (typically when N per group is less than 10)
- For each experiment, you should identify the statistical tests used, exact values of N, definitions of center, methods of multiple test correction, and dispersion and precision measures (e.g., mean, median, SD, SEM, confidence intervals; and, for the major substantive results, a measure of effect size (e.g., Pearson's r, Cohen's d)
- Report exact p-values wherever possible alongside the summary statistics and 95% confidence intervals. These should be reported for all key questions and not only when the p-value is less than 0.05.

Please outline where this information can be found within the submission (e.g., sections or figure legends), or explain why this information doesn’t apply to your submission:

Statistical methods are described in **Material and Methods, p. 25, 724-733**. A table with all statistical information for each figure is provided (**Table 3**).

(For large datasets, or papers with a very large number of statistical tests, you may upload a single table file with tests, Ns, etc., with reference to sections in the manuscript.)

**Group allocation**

- Indicate how samples were allocated into experimental groups (in the case of clinical studies, please specify allocation to treatment method); if randomization was used, please also state if restricted randomization was applied
- Indicate if masking was used during group allocation, data collection and/or data analysis

Please outline where this information can be found within the submission (e.g., sections or figure legends), or explain why this information doesn’t apply to your submission:

All animals were genotyped (ear clip biopsies) and PCRs were performed to distinguish between animals of control, *Dab1* CKO and *Dab1^-/-^* genotypes.

**Additional data files (“source data”)**

- We encourage you to upload relevant additional data files, such as numerical data that are represented as a graph in a figure, or as a summary table
- Where provided, these should be in the most useful format, and they can be uploaded as “Source data” files linked to a main figure or table
- Include model definition files including the full list of parameters used
- Include code used for data analysis (e.g., R, MatLab)
- Avoid stating that data files are “available upon request”

Please indicate the figures or tables for which source data files have been provided:

Table 3 with statistical information also provides numerical data and distributions.

Code used for analysis is provided as a ZIP file.
